# Outcomes in patients with cancer following opioid initiation in Japan: an exploratory study

**DOI:** 10.1186/s13104-026-07752-3

**Published:** 2026-03-05

**Authors:** Takaomi Kessoku, Takahiro Higashibata, Shunsuke Oyamada, Yasuhide Morioka, Yuichi Koretaka, Yasushi Ichikawa, Atsushi Nakajima, Takayuki Hisanaga

**Affiliations:** 1 Department of Palliative Medicine, International University of Health and Welfare Narita Hospital, 852 Hatakeda, Narita, Chiba 286-8520 Japan; 2 Department of Gastroenterology, International University of Health and Welfare School of Medicine, 4-3 Kozunomori, Narita, Chiba 286-8686 Japan; 3 Department of Gastroenterology and Hepatology, Yokohama City University School of Medicine, 3-9 Fukuura, Kanazawa-ku, Yokohama, 236-0004 Japan; 4 Department of Palliative and Supportive Care, Institute of Medicine, University of Tsukuba, 1-1-1 Tennodai, Tsukuba, Ibaraki 305-8575 Japan; 5 Department of Biostatistics, JORTC Data Center, 2-54-6-302, Nishinippori, Arakawa-ku, Tokyo, 116-0013 Japan; 6 Medical Affairs Department, Shionogi & Co., Ltd., GRAND GREEN OSAKA South Building Park Tower, 5-54 Ofuka-cho, Kita-ku, Osaka, 530-0011 Japan; 7 Data Science Department, Shionogi & Co., Ltd., GRAND GREEN OSAKA South Building Park Tower, 5-54 Ofuka-cho, Kita-ku, Osaka, 530-0011 Japan; 8 Department of Oncology, Yokohama City University School of Medicine, 3-9 Fukuura, Kanazawa-ku, Yokohama, 236-0004 Japan; 9 Department of Gastroenterology and Hepatology, International University of Health and Welfare Atami Hospital, 13-1 Higashi-kaigan-cho, Atami, Shizuoka 413-0012 Japan; 10 Department of Palliative Medicine, Tsukuba Medical Center Hospital, 1-3-1 Amakubo, Tsukuba, Ibaraki 305-8558 Japan

**Keywords:** Cancer pain, Nationwide claims database, Opioids, Opioid-induced constipation, Patient outcomes, Real-world

## Abstract

**Objective:**

This cross-sectional study explored outcomes in patients with cancer following opioid initiation (maximum observation period: 6 months), based on an analysis of laxative prescription patterns for opioid-induced constipation (OIC) using the Medical Data Vision Co., Ltd., a nationwide acute care hospital claims database in Japan.

**Results:**

Of 26,939 patients included, 16,214 (60.2%) developed OIC, defined as the prescription of a laxative on or after the fourth day following the initiation of opioid therapy. At baseline, the average age was 69.3 years, with 58.0% being male patients. Overall, 42.7% of patients were hospitalized at the time of opioid initiation (OIC subgroup: 51.1%; non-OIC subgroup: 30.1%). The majority of patients (53.9%) had observation data for less than 6 months (OIC subgroup: 61.8%; non-OIC subgroup: 42.0%). Although there were disparities in patient backgrounds and observation periods, the total healthcare costs incurred were approximately ¥2,400,000, with ¥2,800,000 in the OIC subgroup and ¥1,800,000 in the non-OIC subgroup. Overall, these results highlight the importance of implementing appropriate OIC management strategies in Japan. However, it is noteworthy that the sequence of events between OIC onset and clinical outcomes remains unclear.

## Introduction

Pain is a common and distressing symptom in patients with cancer, and its undertreatment remains a global issue, including in Japan [1]. Opioid analgesics are recommended for the treatment of moderate-to-severe cancer pain [1, 2]. However, their therapeutic benefits may be compromised by undesirable side effects [3], among which opioid-induced constipation (OIC) is of particular concern due to its high prevalence [3, 4].

Studies involving Japanese patients with cancer pain receiving opioids have reported OIC incidence rates of 55–65% [5, 6]. Overall, OIC incidence in patients with cancer is 40–60%, depending on the dose and type of opioid used [7].

Patients with advanced cancer typically have a poor prognosis [8]. The presence of OIC significantly impairs quality of life by causing psychological distress, limiting daily activities, and decreasing work productivity, thereby imposing a substantial burden on patients [9, 10]. Patients with poor overall health receiving opioids may experience severe systemic effects due to OIC [9]. Furthermore, evidence suggests that OIC has a significant impact on healthcare resource utilization, such as outpatient visits, hospitalization, and economic burden, in patients with cancer pain in the United States [11, 12] and other countries [13, 14]. However, the long-term outcomes of patients with cancer pain receiving opioids have not been fully investigated in Japan.

This study aimed to report the outcomes of OIC in patients with cancer following opioid initiation. The analysis used real-world data from Japan, obtained from a previously published study [15].

## Main text

### Study design and data source

This descriptive, exploratory, cross-sectional study was conducted using anonymized data from the same patient cohort as a previously published analysis of laxative prescription patterns for OIC [15]. Briefly, the study utilized a nationwide acute care hospital claims database provided by Medical Data Vision Co., Ltd. (MDV), containing administrative data from 550 hospitals and records from 39 million patients, including those receiving acute inpatient care. MDV collects anonymized administrative data from hospitals, obtains hospital consent, and processes the data into a database. The study period covered opioid initiation from January 2018 to December 2019, thus avoiding the effects of the coronavirus disease 2019 (COVID-19) lockdown, which began in April 2020 in Japan. Patient demographics and outcomes were assessed at the time of opioid initiation and throughout an observation period of up to 6 months.

### Study population

Patients diagnosed with cancer (per the International Classification of Diseases, 10th Revision [ICD-10] codes C00–D09) who initiated opioids and had long-term opioid prescriptions (continuous prescriptions for ≥ 30 days for any opioid; strong opioids: morphine, hydromorphone, oxycodone, fentanyl, tapentadol, methadone, and/or buprenorphine; weak opioids: codeine, tramadol, and/or pentazocine) between January 2018 and December 2019 were included. Continuous prescriptions were defined as opioid prescriptions for ≥ 30 days, including the combination of multiple opioids and irrespective of the purpose of use, with a gap of < 2 days.

Patients prescribed opioids for rescue therapy and those using the buprenorphine transdermal patch, which is not indicated for cancer pain in Japan, were excluded. Patients who had received laxatives within 3 months or opioids within 7 days before opioid initiation were also excluded.

For the analysis, data from eligible patients were presented according to two subgroups: the OIC subgroup and the non-OIC subgroup. The OIC subgroup comprised patients who were prescribed a new laxative on or after the 4th day of opioid initiation, or those who were prescribed a different laxative than the one initially prescribed within the first 3 days. The non-OIC subgroup included patients who were not prescribed any laxatives during the observation period, or those who were prescribed a laxative within the first 3 days of opioid use and did not change their laxative on or after the 4th day, with the laxative prescribed prophylactically to prevent OIC. The overall value was calculated by summing the obtained values for the OIC and non-OIC subgroups.

### Outcomes and assessments

Baseline variables at opioid initiation included age, sex, inpatient/outpatient status, strong/weak opioid type, cancer type, and the presence of peritoneal dissemination. Opioid doses were not considered. During the 6-month observation period from opioid initiation, the following variables were assessed: patient deaths, dropouts (defined as death or absence of medical records after a given time point), incidence of hospitalization (hospitalization frequency, including rehospitalization), opioid type used, total prescription amount of magnesium oxide (amount used per person over 6 months), imaging examinations (computed tomography [CT], magnetic resonance imaging [MRI], and endoscopy; number of times/month), complications (pneumonia, ischemic heart disease, and cerebrovascular disease), and total medical costs (total cost of all medical procedures per person during the observation period).

### Statistical analysis

To explore the outcomes of patients with OIC, descriptive statistics were performed. Continuous variables were reported as mean (standard deviation), while categorical variables were reported as frequencies and proportions. The frequency of imaging examinations was presented as a rate, calculated as the number of examinations per person per month.

## Results

### Patient disposition

Patient disposition was as previously published [15]. Overall, 26,939 patients met the eligibility criteria and were included in the analysis. Of these, 16,214 (60.2%) patients experienced OIC and were included in the OIC subgroup, whereas 10,725 (39.8%) patients did not have OIC and were included in the non-OIC subgroup.

### Patient characteristics at opioid initiation

Patient characteristics such as demographics, malignant disease status, opioid type, and hospitalization status at opioid initiation are summarized in Table 1. Overall, the average age was 69.3 years (OIC subgroup: 69.6 years; non-OIC subgroup: 68.8 years), with 58% being male (OIC subgroup: 59.5%; non-OIC subgroup: 55.9%). Furthermore, 42.7% of patients were hospitalized (OIC subgroup: 51.1%; non-OIC subgroup: 30.1%), and 50.7% used strong opioids (OIC subgroup: 55.6%; non-OIC subgroup: 43.4%). Lung cancer was the most prevalent type of cancer, with an overall frequency of 13.0% (OIC subgroup: 12.6%; non-OIC subgroup: 13.5%). The frequency of peritoneal metastasis was 4.1% (OIC subgroup: 3.9%; non-OIC subgroup: 4.4%).

**Table 1:** Patient baseline characteristics at opioid initiation

Characteristics	Overall^a^ (*N* = 26,939)	OIC subgroup (*n* = 16,214)	Non-OIC subgroup (*n* = 10,725)
Age, years (mean ± SD)	69.3 ± 12.4	69.6 ± 12.2	68.8 ± 12.6
Sex			
Male	15,638 (58.0)	9,644 (59.5)	5,994 (55.9)
Female	11,301 (42.0)	6,570 (40.5)	4,731 (44.1)
Settings			
Inpatient	11,508 (42.7)	8,281 (51.1)	3,227 (30.1)
Outpatient	15,431 (57.3)	7,933 (48.9)	7,498 (69.9)
Opioid type (at initiation)^b^			
Strong opioids	13,661 (50.7)	9,008 (55.6)	4,653 (43.4)
Weak opioids	13,278 (49.3)	7,206 (44.4)	6,072 (56.6)
Cancer type			
Lung cancer	3,496 (13.0)	2,043 (12.6)	1,453 (13.5)
Colon cancer	1,583 (5.9)	728 (4.5)	855 (8.0)
Stomach cancer	1,129 (4.2)	638 (3.9)	491 (4.6)
Breast cancer	1,031 (3.8)	486 (3.0)	545 (5.1)
Uterine cancer	462 (1.7)	275 (1.7)	187 (1.7)
Multiple cancers	6,991 (26.0)	4,335 (26.7)	2,656 (24.8)
Other cancers	12,247 (45.5)	7,709 (47.5)	4,538 (42.3)
Peritoneal dissemination	1,101 (4.1)	627 (3.9)	474 (4.4)

All values are presented as *n*(%), unless otherwise specified

Percentages were calculated using the total number of patients in each subgroup as the denominator

*OIC* opioid-induced constipation,* SD* standard deviation

^a^The overall value was calculated by summing the obtained values for the OIC and non-OIC subgroups

^b^Opioids except codeine, tramadol, and pentazocine were classified as strong opioids

### Patient outcomes during the 6 months after opioid initiation

As shown in Table 2, overall, 53.9% of patients could not be followed up for the entire 6-month period (OIC subgroup: 61.8%; non-OIC subgroup: 42.0%). The overall proportion of mortality was 29.6% (OIC subgroup: 36.5%; non-OIC subgroup: 19.3%), and the incidence of hospitalization increased to 67.1% (OIC subgroup: 78.1%; non-OIC subgroup: 50.5%). The proportion of patients using ≥ 2 opioids was 34.6% (OIC subgroup: 44.1%; non-OIC subgroup: 20.1%), and the total amount of magnesium oxide used was 48,996.1 mg (OIC subgroup: 61,027.1 mg; non-OIC subgroup: 30,807.7 mg). CT scan was the most common imaging examination (OIC subgroup: 0.36 times/month; non-OIC subgroup: 0.25 times/month) (Table 2). The proportion of patients experiencing adverse events after opioid initiation was 30.2% (OIC subgroup: 31.5%; non-OIC subgroup: 28.1%). The average total medical costs per person were ¥2,407,705 (OIC subgroup: ¥2,804,352; non-OIC subgroup: ¥1,808,057) (Table 2).

**Table 2:** Patient outcomes during the 6 months following opioid initiation

Outcomes	Overall^a^ (*N* = 26,939)	OIC subgroup (*n* = 16,214)	Non-OIC subgroup (*n* = 10,725)
Patients lost to follow-up			
At 1 month	1,331 (4.9)	589 (3.6)	742 (6.9)
At 3 months	9,689 (36.0)	6,707 (41.4)	2,982 (27.8)
At 6 months	14,526 (53.9)	10,022 (61.8)	4,504 (42.0)
Incidence of death			
At 1 month	312 (1.2)	170 (1.0)	142 (1.3)
At 3 months	5,371 (19.9)	3,985 (24.6)	1,386 (12.9)
At 6 months	7,984 (29.6)	5,915 (36.5)	2,069 (19.3)
Incidence of hospitalization			
At 1 month	11,523 (42.8)	8,738 (53.9)	2,785 (26.0)
At 3 months	15,950 (59.2)	11,815 (72.9)	4,135 (28.6)
At 6 months	18,076 (67.1)	12,659 (78.1)	5,417 (50.5)
Patients using ≥ 2 opioids	9,314 (34.6)	7,158 (44.1)	2,156 (20.1)
Total amount of magnesium oxide prescribed^b^, mg (mean ± SD)	48,996.1 ± 71,343.0	61,027.1 ± 73,405.5	30,807.7 ± 63,947.4
Frequency of imaging examinations^b^, times/month (mean ± SD)			
CT scan	NC	0.36 ± 0.32	0.25 ± 0.27
MRI	NC	0.08 ± 0.15	0.05 ± 0.12
Endoscopy	NC	0.04 ± 0.10	0.02 ± 0.07
All	NC	0.48 ± 0.43	0.33 ± 0.35
Adverse events			
Pneumonia	4,607 (17.1)	3,029 (18.7)	1,578 (14.7)
Ischemic heart disease	2,887 (10.7)	1,728 (10.7)	1,159 (10.8)
Cerebrovascular disease	2,286 (8.5)	1,372 (8.5)	914 (8.5)
All	8,128 (30.2)	5,109 (31.5)	3,019 (28.1)
Total medical costs^b^, ¥ (mean ± SD)	2,407,705 ± 2,423,149	2,804,352 ± 2,516,155	1,808,057 ± 2,140,141

All values are presented as* n*(%), unless otherwise specified

Percentages were calculated using the total number of patients in each subgroup as the denominator

*CT* computed tomography,* MRI* magnetic resonance imaging,* NC* not calculable,* OIC* opioid-induced constipation,* SD* standard deviation

^a^The overall value was calculated by summing the obtained values for the OIC and non-OIC subgroups

^b^Values are expressed per person

## Discussion

This nationwide acute care hospital claims database analysis assessed OIC outcomes in Japanese patients initiating opioids for cancer pain. Overall, 60.2% of patients experienced OIC. The study is limited in establishing a causal relationship between opioid use and OIC onset. Given the heterogeneity in patient backgrounds and observation periods, a direct comparison may not be appropriate. However, the aggregated data provide insights through numerical comparison.

In the OIC subgroup, a higher proportion of patients initiated strong opioids (55.6%) and were hospitalized (51.1%). The cumulative proportion of patients hospitalized was 67.1% within the first 6 months following opioid initiation. Furthermore, loss to follow-up (61.8%), deaths (36.5%), and hospitalization were higher among patients with OIC during the 6 months after opioid initiation, suggesting that variations in patient backgrounds could be contributing factors. Patients in the OIC subgroup tended to receive ≥ 2 opioids (44.1%), consume more magnesium oxide (61,027.1 mg), and undergo more frequent imaging examinations (0.48 times/month). Additionally, the average total medical costs per person (¥2,804,352) were also higher in the OIC subgroup.

Despite the observed OIC incidence of 60.2%, there were no obvious numerical differences at baseline between the OIC and non-OIC subgroups regarding age, sex, cancer type, or presence of peritoneal metastasis. These findings align with a previous cohort study in Japanese patients with cancer pain who initiated strong opioids without prophylactic laxatives, which reported an OIC incidence of 65% [5]. There was also no significant association between age, sex, or cancer type and the onset of OIC [5].

The present findings suggest a higher proportion of hospitalized patients and those using strong opioids in the OIC subgroup. A previous analysis demonstrated high use of strong opioids among hospitalized patients [15]. Furthermore, a cross-sectional survey showed a higher incidence of hospitalization among patients with cancer pain who received strong opioids [16]; however, it is unclear whether hospitalization or strong opioid use contributes more to OIC onset [16]. In contrast, a previous cohort study in Japan showed no difference in OIC onset between inpatients (54%) and outpatients (46%) [5]. Therefore, strong opioid use may be a more significant factor than hospitalization in OIC onset.

The proportion of patients who could not be followed up for the entire 6-month period was high in the OIC subgroup (61.8%). Since this study used a hospital-derived database, loss to follow-up was likely due to deaths or hospital transfer. Additionally, recorded deaths accounted for nearly half of the loss to follow-up (36.5% in the OIC subgroup). However, this may be an underestimation, as not all deaths are recorded in hospital medical records.

Our aggregated results showed that the incidence of hospitalization was high among patients with OIC (78.1%), with elevated medical costs over the 6-month period, suggesting that hospitalization likely contributed to increased medical costs. Our findings align with the results of an administrative claims database study in the United States that reported a higher cost for pain-related hospitalization in patients with cancer with OIC [11]. Additionally, patients in the OIC subgroup underwent frequent imaging examinations (0.48 times/month), suggesting a more severe condition that likely contributed to high total medical costs.

In this study, a notable proportion of patients with OIC used ≥ 2 types of opioids, suggesting a change in opioids. A cross-sectional study in France reported a change in opioids (7/10 patients) due to constipation or other bowel dysfunction symptoms in patients with cancer pain [16]. Additionally, opioid types may be changed following OIC onset, as constipation frequency varies depending on the opioid type used [7, 17]. In severe cases, oral administration may become difficult, necessitating a change in the administration route.

In this study, OIC onset was defined based on laxative prescription. The large amount of magnesium oxide prescribed to the non-OIC subgroup suggests that many patients without OIC received magnesium oxide prophylactically (within 3 days of opioid initiation) to prevent OIC onset. This study specifically investigated magnesium oxide as a laxative; however, the study population also received naldemedine after magnesium oxide, as previously reported [15]. Therefore, patients who received naldemedine within ≤ 3 days of opioid initiation and did not change it were likely included in the non-OIC subgroup.

## Limitations

This study database includes data from hospitals with acute inpatient care, excluding small hospitals and clinics, and does not track medications prescribed at other hospitals or over-the-counter drug use. Clinical symptoms, constipation severity, patient condition, and opioid dose were not considered. OIC was identified based on laxative prescriptions rather than clinical diagnosis. The presence or absence of prophylactic laxative administration was not considered. Patient backgrounds were not matched, limiting direct comparisons between the OIC and non-OIC subgroups. Furthermore, many dropouts were included in the analysis. Since the OIC subgroup had more inpatients at opioid initiation, it is possible that their prognosis or performance status was poorer at opioid initiation. It was also not possible to distinguish hospitalization type (unplanned vs. scheduled, such as for inpatient chemotherapy). Lastly, the temporal relationship between OIC onset and each outcome remains unclear, as some patients may have been hospitalized before OIC onset. Despite these limitations, it has been reported that 56% of patients with cancer without prior constipation developed OIC early (within 2 weeks) after initiating opioids [5], and OIC onset in this study aligns with previous findings, suggesting that OIC onset may have been appropriately captured. Future studies incorporating matched patient background factors and clinical information are needed to validate these hypotheses.

In conclusion, these results provide foundational information for developing appropriate OIC management strategies in Japan.

## Data Availability

The database provided by Medical Data Vision Co., Ltd. was used under a license agreement. The datasets used and/or analyzed during the current study are available from the corresponding author on reasonable request with permission from Medical Data Vision Co., Ltd.

## Abbreviations

COVID-19: Coronavirus disease 2019
CT: Computed tomography
ICD-10: International Classification of Diseases, 10th Revision
MDV: Medical Data Vision
MRI: Magnetic resonance imaging
OIC: Opioid-induced constipation
